# The hidden cost of specialization: a mixed-methods study on burnout, mental health, and financial ınstability in dental trainees

**DOI:** 10.1186/s12909-026-08584-2

**Published:** 2026-01-12

**Authors:** Süha Alpay, Elif Cakılkaya, Burcu Dagdelen, Dilara Arslan

**Affiliations:** Department of Endodontics, Faculty of Dentistry, Istanbul Aydın University, Istanbul, Türkiye

**Keywords:** Burnout, Postgraduate dental education, Mental health, Mixed-methods research, Dental trainee

## Abstract

**Background:**

Burnout is a significant issue in postgraduate dental education, exacerbated by demanding clinical duties, academic pressures, and insufficient resources. This cross-sectional survey incorporated an embedded qualitative component analyzed using inductive thematic analysis.

**Methods:**

This cross-sectional survey incorporated an embedded qualitative component analyzed using inductive thematic analysis. Participants filled out validated tests for burnout (OLBI-SS), mental health symptoms (PHQ-4), perceived stress (PSS-4), resilience (BRS), and perceived institutional and supervisory support. We used multiple linear regression to find factors that could lead to burnout. Inductive content analysis was used to put qualitative data from open-ended responses into context with quantitative findings. This study aimed to determine the prevalence of burnout and to examine its individual, supervisory, and institutional correlates among postgraduate dental trainees, using a cross-sectional design with an embedded qualitative component.

**Results:**

Based on a mean OLBI-SS item score ≥3.0, the prevalence of high burnout was 75.4% among postgraduate dental trainees. Burnout levels were high, accompanied by elevated rates of positive screening for anxiety (53.3%) and depressive symptoms (50.0%). In the multivariable model, higher mental health symptoms (β = +1.25, *p =* 0.001) and lower institutional support (β = –3.15, *p =* 0.002) significantly predicted burnout. While gender was not significant in bivariate analysis, male gender emerged as a strong independent predictor (β = +3.61, *p =* 0.041) after controlling for anxiety and depressive symptoms, indicating a suppressor effect where general distress masked specific risks in male trainees. Qualitative analysis revealed four major stressors: financial instability, institutional uncertainty, advisor communication difficulties, and conflicts between clinical and academic responsibilities.

**Conclusion:**

Burnout among dental trainees is caused by a combination of mental health problems, lack of support, and stressors in their environment. Our findings indicate that the quality of the educational environment and the frequency of advisor meetings are more significant determinants of well-being than clinical workload volume alone. Interventions should prioritize organized supervision, clear organization, and financial help.

**Supplementary Information:**

The online version contains supplementary material available at 10.1186/s12909-026-08584-2.

## Background

Burnout is becoming a bigger problem in medical and dental education, especially in postgraduate clinical training settings where academic, clinical, and financial pressures all come together. Burnout, marked by emotional exhaustion, disengagement, and reduced professional efficacy, inflicts significant repercussions on both learners and institutions, as well as on patient care [1, 2]. An increasing amount of evidence shows that health professions trainees are more likely to have symptoms of depression and anxiety than other students. This is likely due to the stress of having to do a lot of schoolwork and care for complex patients [3, 4]. Dental trainees seem especially vulnerable in this regard, as research consistently indicates heightened levels of stress, emotional exhaustion, and academic pressure during specialty and doctoral training [5, 6].

Various factors have been associated with the onset of burnout among healthcare trainees. Individual determinants, such as perceived stress, depressive and anxiety symptoms, and diminished resilience, have been demonstrated to significantly influence the risk of burnout [7–9]. However, modern frameworks place a growing emphasis on the significant impact of the training environment. The Job Demands–Resources (JD-R) model posits that burnout arises not solely from excessive demands but from a disparity between those demands and the resources available to manage them, including supervision quality, feedback, autonomy, and institutional support [10, 11]. In this regard, burnout signifies both an individual and systemic challenge.

Postgraduate dental education presents unique stressors that may increase susceptibility to burnout. Trainees must manage substantial clinical responsibilities alongside demanding academic obligations, encompassing research, thesis development, and laboratory duties. This dual burden, observed across various training systems, generates a persistent strain that may surpass the resilience of individual coping mechanisms [12]. Financial pressure is another factor that is becoming more important. In many countries, including Türkiye, postgraduate dental trainees are in a difficult position: they do the same clinical work as full-time employees but are still considered students. In many settings, postgraduate dental trainees perform extensive clinical duties while receiving limited or no financial compensation and simultaneously bearing the costs of their education [13, 14]. Additionally, institutional and supervisory elements—such as inconsistent mentorship, insufficient feedback, academic disarray, ambiguous progression criteria, and instances of mistreatment—can compromise psychological safety and intensify burnout [15–17].

This study aims to fill these gaps by using a mixed-methods approach to look at burnout among postgraduate dental trainees. This group is specifically defined as PhD and residency students who have a lot of clinical and academic work to do at the same time. Burnout among postgraduate dental trainees represents a growing challenge for workforce sustainability and aligns with global priorities on mental health and well-being, including Sustainable Development Goal 3.

Accordingly, this study aimed to determine the prevalence of burnout and to examine its individual, supervisory, and institutional correlates using a cross-sectional survey with an embedded qualitative component.

Even though these issues are getting more attention, burnout among postgraduate dental trainees is still not well understood compared to medical residency populations. Only a limited number of studies have employed mixed-methods designs to simultaneously quantify burnout and explore lived experiences in postgraduate dental trainees [5, 10]. Furthermore, there is a paucity of research investigating the combined effects of mental health symptoms, resilience, perceived support, and financial or institutional uncertainty within a singular analytical framework.

Therefore, this mixed-methods study aimed to examine the prevalence of burnout among postgraduate dental trainees and to explore its individual, supervisory, and institutional correlates by integrating validated quantitative measures with qualitative analysis of lived experiences.

## Materials and methods

### Study design and setting

This study employed a cross-sectional survey design incorporating an embedded qualitative component analyzed using inductive thematic analysis. The survey was conducted at a university-based dental faculty in Türkiye, which provides various specialty and PhD programs. From June 15, 2025, to November 15, 2025, data were gathered online through a secure survey platform. The research complied with the tenets of the Declaration of Helsinki.

### Participants and procedure

Eligible participants consisted of postgraduate dental trainees enrolled in PhD or specialization programs in endodontics, prosthodontics, orthodontics, periodontology, restorative dentistry, and oral and maxillofacial surgery. The criteria for inclusion were being actively enrolled in a postgraduate program and giving informed consent. A strategy of convenience sampling was used. Participation was voluntary and anonymous, and all participants provided electronic informed consent prior to data collection.

### Study

The sample size was determined a priori using power analysis to ensure adequate statistical power for detecting medium effect sizes.

### Sample size and power analysis

G*Power 3.1 was used to do an a priori power analysis. The effect size was determined to be 0.35, predicated on expected medium-sized correlations among principal study variables (r = 0.30–0.40). With an alpha level of 0.05 and a desired power of 0.80, the minimum sample size needed was 52 people.

### Measures

#### Variables

The primary outcome variable was burnout, measured using the OLBI-SS total score. Independent variables included psychological distress (PHQ-4), perceived stress (PSS-4), psychological resilience (BRS), perceived institutional and supervisory support, gender, age, funding status, and academic characteristics. All variables were selected a priori based on theoretical relevance within the Job Demands–Resources framework.

Participants’ age, gender, department, phase of doctoral training (coursework vs. thesis), weekly clinical hours, frequency of advisor meetings, and funding status (self-funded vs. scholarship) were recorded. To evaluate psychological well-being and environmental factors, the following validated instruments were employed:

Burnout: Burnout levels were assessed using the 10-item Oldenburg Burnout Inventory–Student Version (OLBI-SS) developed by Demerouti et al. [18]. The inventory evaluates two core dimensions: exhaustion (physical and cognitive fatigue) and disengagement (distancing oneself from one's studies). Items are rated on a 5-point Likert scale. A total score was calculated for the analyses, where higher scores indicate greater levels of burnout. High burnout was defined as a mean OLBI-SS item score of ≥3.0, consistent with previous studies using the student version of the Oldenburg Burnout Inventory.

Perceived Stress: The 4-item Perceived Stress Scale (PSS-4) was utilized to assess stress perceptions related to lack of control and excessive demands, with response options ranging from 0 (never) to 4 (very often). A brief form of the original scale developed by Cohen et al. [19] and shortened by Cohen [20]. We used the Turkish adaptation validated by Eskin et al. [21].

Anxiety and Depression: Psychological distress was assessed using the Patient Health Questionnaire-4 (PHQ-4), which comprises two items measuring anxiety [Generalized Anxiety Disorder – 2 (GAD-2)] and two items measuring depressive symptoms (PHQ-2) [22]. For the Turkish context, the validity of the items was grounded in the Turkish validation study of the GAD-7 scale [23]. Total scores range from 0 to 12. Scores of 3 or higher on either the GAD-2 or PHQ-2 subscales were considered indicative of clinically relevant symptoms.

Resilience: Psychological resilience was assessed using the Brief Resilience Scale (BRS) developed by Smith et al. [24]. The Turkish adaptation and validity study of the scale were conducted by Doğan [25]. The scale consists of six items rated on a 5-point Likert scale. After reverse-coding negatively worded items (items 2, 4, and 6), a mean score is calculated. Higher scores indicate greater resilience. Based on the established cut-off values, scores are interpreted as follows: 1.00–2.99 indicates low resilience, 3.00–4.30 indicates normal resilience, and 4.31–5.00 indicates high resilience.

Perceived Institutional and Supervisory Support: A 5-item scale was created for this study to evaluate perceived institutional and supervisory support, focusing on academic guidance, clarity of expectations, supervisor availability, feedback quality, and perceived mental health support from the institution. Each item was scored using a 5-point Likert scale. The Perceived Institutional and Supervisory Support scale was developed specifically for the purposes of this study to capture key aspects of the training environment, including academic guidance, clarity of expectations, supervisor accessibility, feedback quality, and perceived institutional mental health support. While the scale has not undergone prior external validation, its internal consistency was assessed in the present sample using Cronbach’s alpha. The Perceived Institutional and Supervisory Support scale was developed specifically for this study. Although the scale has not undergone prior external validation, internal consistency was acceptable in the present sample (Cronbach’s α = 0.74).

#### Qualitative data collection

To investigate the contextual factors contributing to burnout, the survey incorporated an open-ended question aimed at eliciting trainees’ subjective experiences: “*What is the situation that challenges you the most or makes you feel burned out during the doctoral process*?”.

#### Statistical methods

A mix of Python (Pandas) and SPSS v27 was used to preprocess the data. Prior to analysis, categorical responses were transformed into numerical values, and reverse-coded items for the BRS and PSS-4 were appropriately recoded. Pairwise deletion was used to deal with missing data.

We used IBM SPSS Statistics v27 for statistical analyses, and we also used Python-based validation procedures. We summarized continuous variables as mean ± standard deviation and categorical variables as frequencies. We used Cronbach's α to check the internal consistency of each scale. The Shapiro–Wilk test was used to look at distributional assumptions.

We used Pearson or Spearman correlation coefficients to look at the relationships between burnout, psychological measures, and how often students met with their advisors. However, we mostly used Spearman's rank correlations because the distributions were not normal. Mann–Whitney U tests were used to look at differences between groups based on gender, funding status, and training stage.

A multiple linear regression model was developed to ascertain independent predictors of burnout, utilizing the OLBI total score as the dependent variable and incorporating PSS-4, PHQ-4, BRS, perceived support, gender, age, and funding status as predictor variables. Even though the sample size was small compared to the number of predictors, all of the variables were kept in the model because they were theoretically important to the JD-R model, not just because of statistical selection. Statistical significance was established at *p* < 0.05, and regression coefficients were presented alongside 95% confidence intervals.

Given the sample size (*N=*60) relative to the number of predictors in the regression model, stringent diagnostic evaluations were underscored to guarantee the model's stability. A post-hoc analysis confirmed that the sample size was sufficient, providing adequate power (>0.80) to detect the substantial effect sizes noted (R^2^ = 0.489). The low Variance Inflation Factors (VIF < 2.0) and the normal distribution of residuals also showed that the regression estimates were strong and not artificially high because of multicollinearity or outliers.

Inductive thematic analysis was used to look at the qualitative responses. We looked at all the stories, came up with some initial codes, and then grouped those codes into bigger themes, such as financial instability, communication between supervisors, uncertainty in the system, and clinical workload. The final themes were combined with the quantitative results to make a full mixed-methods interpretation.

Due to the sample size (*N* = 60) relative to the number of predictors, stringent diagnostic checks were prioritized to ensure model stability. Bootstrapping was used to evaluate the stability and robustness of regression coefficients given the relatively small sample size. Specifically, bootstrapping procedures with 1,000 and 5,000 resamples were applied, and bootstrap confidence intervals were examined to assess the consistency of significant predictors (see Supplementary File S1). Predictor variables were retained in the regression model based on theoretical relevance within the Job Demands–Resources framework rather than stepwise or data-driven selection procedures.

#### Qualitative analysis

Responses to the open-ended question were analyzed using inductive thematic analysis. All responses were systematically reviewed and coded independently. Initial codes were subsequently refined through iterative review, and final themes were generated through repeated examination and were integrated with the quantitative findings to provide contextual depth. Qualitative coding was conducted by a single researcher; the use of plural phrasing reflects academic convention rather than multiple independent coders.

#### Bias

Potential sources of bias include the self-reported nature of the survey data and the single-center study design. To mitigate these risks, participation was anonymous, standardized and validated instruments were used, and qualitative findings were triangulated with quantitative results to enhance interpretive credibility.

## Results

### Participant and academic characteristics

The survey was distributed to all 65 eligible postgraduate trainees in the faculty. A total of 60 trainees participated, yielding a response rate of 93%. The mean age was 27.2 ± 2.5 years, and 58 % were female. Trainees represented multiple specialties, including endodontics, prosthodontics, orthodontics, and periodontology, and the majority (70%) were in the coursework phase of their doctoral programs. Most students were self-funded (88.3%), while a small minority received institutional or external scholarships (11.7%). Demographic and academic characteristics are summarized in Table 1.

**Table 1:** Demographic characteristics of the participants (*N* = 60)

**Characteristic**	**Category**	**n**	**%**
Age	Mean ± SD	60	27.2 ± 2.5
Gender	Female	35	58.3
	Male	25	41.7
Education Phase	Coursework	42	70
	Thesis	18	30
Financial Status	Self-funded	53	88.3
	Scholarship	7	11.7

### Burnout, stress, mental health, resilience, and support

This subsection summarizes the descriptive findings related to burnout, perceived stress, mental health symptoms, psychological resilience, and perceived institutional and supervisory support. Participants reported moderate-to-high levels of burnout, with a mean OLBI-SS total score of 33.5 ± 7.0. Using established OLBI-SS cut-off values, 75.4% of participants met the criteria for high burnout, indicating a substantial burden in this population.

Perceived stress levels were elevated (PSS-4 mean 8.9 ± 2.37), and mental health symptoms were prominent (PHQ-4 mean 5.98 ± 2.66), with 53.3% and 50.0% of participants screening positive for anxiety and depressive symptoms, respectively; 20.0% reported severe symptom levels. Psychological resilience was moderate (mean 3.36 ± 0.84), whereas perceived institutional and supervisory support was low (mean 2.74 out of 5).

Internal consistency was satisfactory across all instruments, with Cronbach’s α values of 0.78 for the OLBI-SS, 0.72 for the PSS-4, 0.79 for the PHQ-4, 0.75 for the BRS, and 0.74 for the Perceived Institutional and Supervisory Support scale. Table 2 presents the mean scores for psychological well-being measures and the prevalence of anxiety and depressive symptoms among participants.

**Table 2:** Descriptive statistics for psychological and academic well-being measures

**Variable**	**Mean ± SD**	**n (%)**
Burnout (OLBI-SS)	33.50 ± 7.00	–
Perceived Stress (PSS-4)	8.90 ± 2.37	–
Mental Health Symptoms (PHQ-4 Total)	5.98 ± 2.66	–
• Positive Screening for Anxiety	–	32 (53.3%)
• Positive Screening for Depression	–	30 (50.0%)
• Severe Symptom Levels	–	12 (20.0%)
Psychological Resilience (BRS)	3.36 ± 0.84	–
Institutional/Supervisory Support	2.74 ± 0.81*	–

All continuous variables are reported as mean ± standard deviation. Cronbach’s α values: OLBI-SS = 0.78; PSS-4 = 0.72; PHQ-4 = 0.79; BRS = 0.75; Perceived Institutional and Supervisory Support = 0.74

### Correlations between burnout and psychological/contextual variables

Correlation analyses revealed significant associations between burnout and key study variables. Pearson correlation coefficients (r), reported as effect size measures, along with corresponding p-values, are presented in Table 3. Higher levels of perceived stress (r = 0.45, *p* < 0.001) and depressive/anxiety symptoms (r = 0.55, *p* < 0.001) were associated with increased burnout, while psychological resilience was inversely correlated with burnout (r = –0.31, *p =* 0.015). In terms of institutional factors, both perceived institutional support (r = –0.39, *p =* 0.003) and the frequency of advisor meetings (r = –0.32, *p =* 0.012) were inversely associated with burnout. Burnout was not significantly correlated with age, study year, or weekly clinical hours (all *p* > 0.05). No significant gender differences were observed in bivariate analyses, although gender effects were subsequently identified in multivariable modeling. Bivariate correlations between burnout scores and key psychological and demographic variables are presented in Table 3.

**Table 3:** Correlations between burnout and key study variables

**Variable**	**r (effect size)**	***p*-value**
Perceived Stress	0.45	<0.001
Depressive/Anxiety Symptoms (PHQ-4)	0.55	<0.001
Psychological Resilience	−0.31	0.015
Perceived Institutional Support	−0.39	0.003
Age	−0.08	0.54
Study Year	0.06	0.63
Weekly Clinical Hours	0.04	0.74
Advisor Meeting Frequency	−0.32	0.012

Pearson correlation coefficients (r) are reported as effect sizes. Effect sizes were interpreted as small (r ≈ 0.10), moderate (r ≈ 0.30), and large (r ≥ 0.50). All *p*-values are two-tailed

### Burnout Differences by perceived support level

Participants perceiving low institutional/supervisory support (<3) demonstrated substantially higher burnout scores (34.94 ± 7.00) compared to those reporting moderate-to-high support (31.35 ± 6.68; *p =* 0.027). Furthermore, the low-support group reported significantly higher levels of anxiety and depressive symptoms (PHQ-4 score: 6.35 vs. 5.43; *p =* 0.011), as detailed in Table 4.

**Table 4:** Comparison of burnout and mental health scores by perceived institutional support level

Variable	Low Support (< 3)(Mean ± SD)	Moderate-to-High Support (≥ 3)(Mean ± SD)	*p*-value
Burnout Score (OLBI-SS)	34.94 ± 7.00	31.35 ± 6.68	0.027*
Mental Health Symptoms (PHQ-4)	6.35 ± 2.56	5.43 ± 2.78	0.011*

### Predictors of burnout

A multiple linear regression analysis was conducted to identify independent predictors of burnout. The model demonstrated strong explanatory power, with an adjusted R2 of 0.423, indicating that the included predictors accounted for approximately 42% of the variance in burnout levels.

In the multivariable analysis, perceived stress, resilience, clinical workload, funding status, and age were not significant independent predictors of burnout. Although gender was not associated with burnout in bivariate analyses, male gender emerged as a significant predictor in the adjusted model, corresponding to higher burnout scores (β = 3.61, *p =*.041). A subgroup trend suggested higher burnout among scholarship recipients; however, the number of participants in this group was small (*n* = 6). Independent predictors identified in the regression model are presented in Table 5. Bootstrap analyses supported the stability of the regression coefficients (see Supplementary File 1).

**Table 5:** Multiple linear regression model predicting burnout levels

**Predictor**	**β (Coefficient)**	**SE**	**95% CI**	***p*-value**
Mental Health Symptoms (PHQ-4)	+1.25	0.37	0.52 to 1.98	0.001
Institutional/Supervisory Support	–3.15	0.93	–5.02 to –1.28	0.002
Male Gender (Ref: Female)	+3.61	1.72	0.14 to 7.08	0.041

R^2^ = 0.489, Adjusted R^2^ = 0.423. “ns” = not significant (*p* > 0.05)

Subgroup analyses were conducted to explore potential differences in burnout levels across selected participant characteristics, including funding status and gender. These analyses were exploratory in nature and performed descriptively due to the limited sample size of certain subgroups, particularly scholarship recipients. Therefore, subgroup findings should be interpreted cautiously and are presented as hypothesis-generating observations rather than confirmatory results.

### Qualitative findings: stressors and lived experiences

A total of **52 trainees** provided open-ended responses elaborating on the most challenging aspects of their doctoral experience. The thematic analysis revealed that financial instability was the most prevalent stressor, with trainees describing the heavy burden of self-financed training; one participant noted, “Supervisors act as if everyone comes from wealth… not realizing we need to make a living causes significant stress” (Participant 18). This financial strain was often compounded by institutional uncertainty and disorganization, where students described the environment as chaotic and unpredictable, highlighting “the constant uncertainty about everything… not knowing what will happen next” (Participant 11). Additionally, academic challenges were prominent, including advisor communication problems (11%)—characterized by feelings of isolation and unreachable supervisors—and thesis-related pressure (9%), where high research expectations were intensified by simultaneous clinical responsibilities (Table 6).

**Table 6:** Themes Identified from the Open-Ended Question

**Theme**	**Description**	**Representative Quote**
Financial Instability	Economic burden of self-funded postgraduate training; perceived financial insecurity	“Not everyone is born into luxury… financial pressure is overwhelming.”
Institutional Uncertainty & Disorganization	Unclear processes, inconsistent expectations, unpredictable academic environment	“You never know what will happen next. The lack of clarity is exhausting.”
Advisor Communication Problems	Limited feedback, irregular meetings, difficulty accessing supervisors	“I feel alone in the thesis process; my advisor is unreachable.”
Thesis-Related Pressure	Stress due to research expectations, lack of structured guidance	“The thesis workload is overwhelming without proper guidance.”
Clinical–Academic Conflict	Challenges balancing clinical duties with academic responsibilities	“Clinical and academic roles constantly clash.”
Psychological Pressure/Mobbing	Experiences of intimidation, harsh communication, emotional distress	“Sometimes the way we are spoken to feels humiliating.”
Time-Management Strain	Difficulty managing simultaneous clinical, academic, and personal demands	“Everything is urgent, and I feel like I’m constantly behind.”

### Summary of integrated findings

Quantitative analyses demonstrated associations between burnout, mental health symptoms, and perceived institutional support. Qualitative analysis identified recurrent themes related to financial strain, supervisory communication, organizational uncertainty, and clinical–academic conflict. These findings were examined together within the mixed-methods framework.

**Fig. 1:**
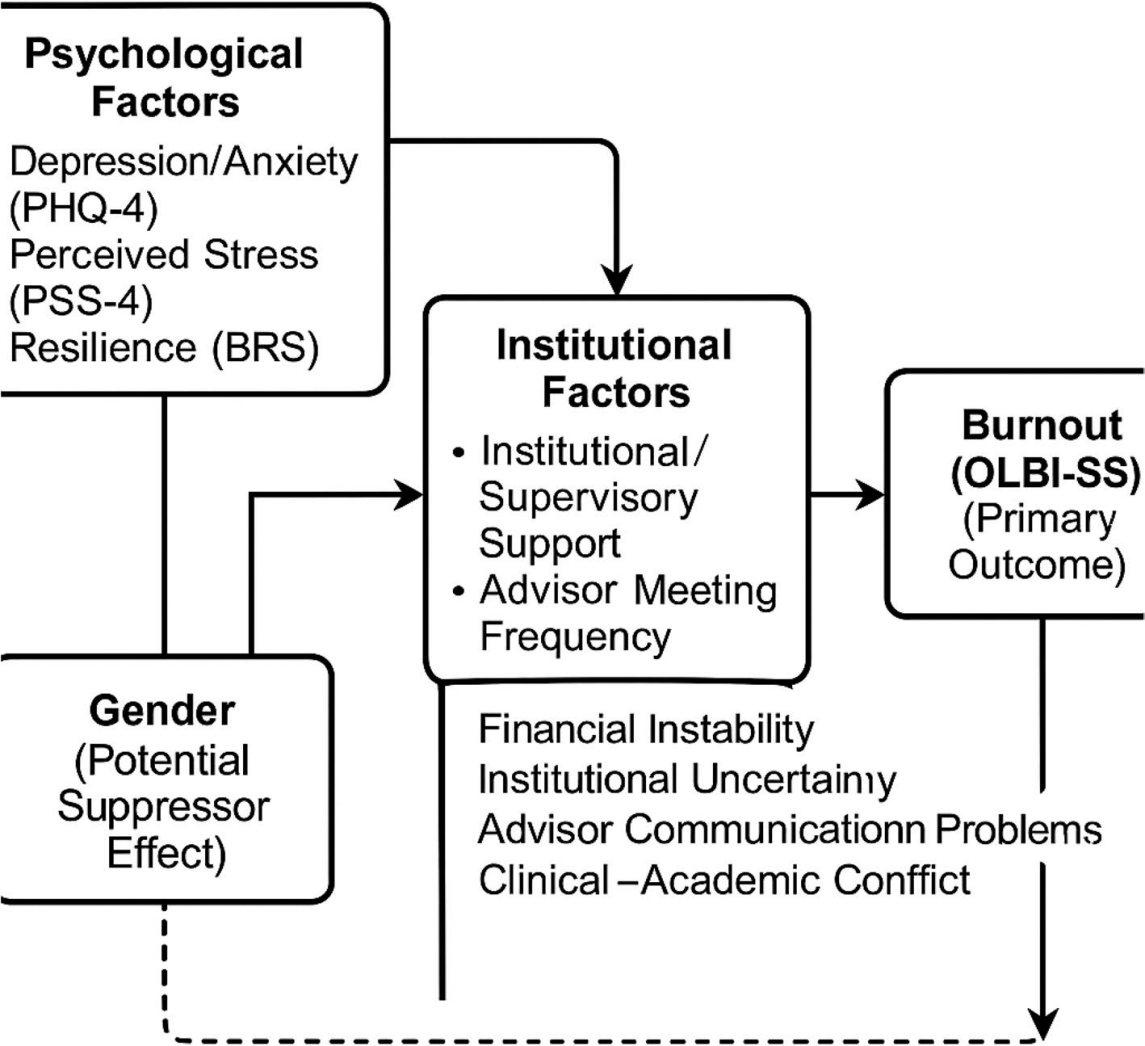
Conceptual Framework for Burnout Among Postgraduate Dental Trainees

Figure 1 illustrates the proposed conceptual model integrating psychological factors (PHQ-4, PSS-4, resilience), institutional characteristics (supervisory support, advisor meeting frequency, organizational clarity), and contextual stressors (financial instability, institutional uncertainty, communication problems, clinical–academic conflict) contributing to burnout among postgraduate dental trainees. Gender emerged as a potential suppressor effect in the multivariable model***.***

## Discussion

This mixed-methods study examined burnout among postgraduate dental trainees by integrating quantitative indicators of psychological distress and institutional factors with qualitative accounts of lived experience. The findings indicate that burnout in this population is not merely a function of workload, but reflects a complex interplay between mental health symptoms, supervisory support, and broader institutional and financial stressors. Notably, more than three-quarters of the trainees (75.4%) met criteria for high burnout, underscoring the substantial psychological burden associated with postgraduate dental training. These rates exceed those typically reported in the general population and align with global evidence identifying dental trainees as a particularly vulnerable group within health professions education.

A striking finding of this study is the high prevalence of psychological distress, with 53.3% of participants screening positive for anxiety and 50% for depressive symptoms. Consistent with prior research among dental and health professions trainees [26, 27]. The strong association between burnout and mental health symptoms, and the identification of PHQ-4 as the strongest predictor of burnout in multivariable analyses, reinforces ongoing discussions regarding the overlap between burnout and psychological symptom burden in high-intensity training environments. Although burnout and depressive symptoms are conceptually distinct constructs, increasing evidence suggests substantial phenomenological overlap under conditions of chronic occupational stress. In the present study, mental health symptoms were assessed using the PHQ-4 as a brief screening instrument designed to capture general psychological distress rather than to establish clinical diagnoses. Accordingly, the observed associations should be interpreted as reflecting co-occurring symptom burden and psychological distress rather than clinically diagnosed anxiety or depressive disorders. This interpretation is supported by meta-analytic evidence indicating shared biological and environmental mechanisms underlying burnout and depressive symptomatology [28]. Unlike medical residents who often share responsibility within large teams, dental trainees typically assume early and largely solitary responsibility for irreversible surgical procedures. This combination of technical precision, individual accountability, and evaluative pressure may contribute to sustained sympathetic activation and the elevated levels of emotional exhaustion observed in this population [29]. Accordingly, these findings should be interpreted as reflecting co-occurring psychological distress rather than clinically diagnosed depressive or anxiety disorders.

One of the most novel and statistically significant contributions of this study is the finding regarding gender differences. While bivariate analyses showed no significant difference in burnout levels between male and female trainees, male gender emerged as a strong and independent predictor of higher burnout scores (ß= +3.61) in the multivariable regression model. This emergence only after adjusting for psychological distress (PHQ-4) represents a classic suppressor effect. It suggests that the high levels of reported distress—more commonly expressed by female trainees—may mask the underlying burnout risk in males. When distress levels are held constant in the multivariate model, the specific vulnerability of male trainees, likely driven by depersonalization and cultural stoicism, becomes statistically visible and unmasks a hidden risk group often overlooked in simple descriptive studies. Although literature frequently reports higher emotional exhaustion in females, recent meta-analyses suggest that males may experience burnout differently, predominantly through depersonalization and cynicism rather than emotional expression [30]. One of the most novel findings of this study concerns gender differences in burnout. While no significant differences were observed in bivariate analyses, male gender emerged as an independent predictor of higher burnout scores after adjustment for psychological distress, representing a classic suppressor effect. This suggests that distress levels—more frequently expressed by female trainees—may mask burnout risk among males in unadjusted analyses.

Previous research indicates that men may experience burnout predominantly through depersonalization and cynicism rather than emotional expression [30]. Within the Turkish sociocultural context, expectations related to financial responsibility, emotional restraint, and rapid career stability may intensify burnout risk among male trainees during prolonged postgraduate education. Consistent with prior literature, men may also be less likely to seek psychological support, potentially delaying recognition of distress until burnout becomes pronounced [31]. Additionally, within the cultural context of Türkiye, societal expectations for men to quickly achieve career stability may intensify the stress caused by the prolonged financial uncertainty and ambiguous status inherent in doctoral training [5].

Our study found no significant correlation between weekly clinical hours and burnout scores, contradicting the view that burnout is driven solely by workload volume. Rather, the evidence indicates that the structural and supportive aspects of the training setting are significantly more crucial for the well-being of trainees. This finding is strongly backed by the Job Demands-Resources (JD-R) Model [32]. This theoretical framework suggests that high job demands, such as workload, can be manageable and even inspiring if they are paired with adequate resources.

However, when resources are scarce—as indicated by the low institutional support scores in our study, even moderate demands can lead to severe burnout [32]. Our qualitative data corroborates this quantitative finding; participants frequently cited systemic issues such as organizational chaos, uncertainty, and lack of clear criteria rather than clinical fatigue. This aligns with Dyrbye et al. [33], who argued that reducing duty hours alone is insufficient to prevent burnout without addressing the underlying educational climate.

Contrary to the assumption that burnout is driven primarily by workload volume, weekly clinical hours were not significantly associated with burnout levels. Instead, findings emphasize the critical role of structural and supportive components of the training environment. Within the Job Demands–Resources framework, job demands may be tolerated when balanced by adequate resources such as supervision, feedback, and organizational clarity.

Both the quality of perceived supervisory support and the frequency of advisor meetings were inversely associated with burnout. Regular and structured supervision may reduce the uncertainty inherent in doctoral training, enhance perceived control, and buffer the adverse effects of chronic stress. These findings align with prior research demonstrating the protective role of effective mentorship in academic medicine [34, 35]. Conversely, low-support environments characterized by absentee supervision may exacerbate burnout and psychological distress [34].

A critical contextual factor in our study is that 89.5% of the participants were self-funded, and financial instability emerged as the most frequent stressor in the qualitative analysis. This highlights a structural vulnerability in the postgraduate education system in many developing economies. Dental trainees are often adults in their late 20 s who face the dissonance of being highly skilled professionals yet remaining financially dependent or precarious. El-Ghoroury et al. identified financial debt as one of the top stressors contributing to anxiety among graduate psychology students [36]. In a similar vein, Woolf et al. [37] found that financial debt among medical and dental students is closely associated with diminished academic performance and reduced psychological well-being. While our quantitative analysis did not reveal a statistically significant difference related to funding status, this may reflect limited statistical power related to the small size of the scholarship subgroup (*n=*7). However, the qualitative evidence unequivocally points to financial strain as a core component of the burnout syndrome in this population. This discrepancy highlights the critical value of the mixed-methods design: the qualitative narratives captured a severe stressor that quantitative statistical power alone failed to detect due to sample constraints [37]. It should be noted that financial strain was captured quantitatively only through a dichotomous funding status variable (self-funded vs. scholarship-supported), which may not fully reflect the complexity, severity, or day-to-day impact of financial stress experienced by trainees. As such, the quantitative findings may underestimate the contribution of financial strain to burnout, while the qualitative data provided a more nuanced and detailed account of this stressor.

The results of this research have immediate consequences for dental schools and decision-makers focused on developing a sustainable academic workforce. Supervision should not be left to personal choice; institutions need to establish compulsory, organized progress meetings to guarantee ongoing feedback and social support. By acknowledging the silent risk among male trainees, institutions should implement proactive, non-stigmatizing mental health programs that encourage help-seeking behaviors across all genders. Additionally, interventions should extend beyond individual resilience training and concentrate on organizational ergonomics—clarifying role responsibilities, ensuring clear thesis criteria, and addressing the financial instability of trainees where feasible [33]. Furthermore, the triangulation of the lack of organizational support identified in quantitative analysis with the themes of chaos and uncertainty highlighted in the qualitative findings strengthens the reliability of the results, thus balancing potential deviations due to the sample size.

A key strength of this study lies in the integration of quantitative findings with an embedded qualitative component, which provided contextual support for the statistical results. Although the qualitative component was limited to a single open-ended question, it offered complementary insights into trainees’ perceived stressors and institutional challenges, thereby enriching the interpretation of the quantitative findings without overstating the depth of qualitative analysis.

Several limitations should be considered when interpreting these findings. The cross-sectional design precludes causal inferences regarding the directionality of observed associations, and the single-center setting may limit generalizability to other postgraduate dental training environments. In addition, subgroup analyses (e.g., by gender and funding status) were exploratory in nature and likely underpowered, and therefore should be interpreted as hypothesis-generating rather than confirmatory. Nevertheless, the study is strengthened by a high response rate, the use of validated psychological screening instruments, and rigorous analytical procedures, including diagnostic checks and bootstrap validation, which support the stability and robustness of the findings despite the modest sample size.

## Conclusion

In conclusion, this study demonstrates a high prevalence of burnout among postgraduate dental trainees, with more than three-quarters of participants meeting criteria for high burnout. Mental health symptoms were common, with substantial proportions of trainees screening positive for anxiety and depressive symptoms, accompanied by elevated levels of perceived stress. Psychological resilience was moderate, while perceived institutional and supervisory support was generally low. Together, these findings highlight a considerable psychological burden during postgraduate dental training.

Postgraduate dental training represents a period of heightened vulnerability in which academic demands intersect with systemic and organizational challenges. Burnout in this population appears to reflect a complex interaction between psychological distress, insufficient supervisory support, organizational ambiguity, and financial insecurity rather than workload volume alone. In addition, financial stress was not measured using a multidimensional quantitative instrument, and reliance on funding status alone may have limited the ability to capture the full scope of financial insecurity. Future studies would benefit from incorporating validated financial stress or debt-related measures to better quantify this important contributor to trainee well-being. To promote a sustainable academic workforce, institutions in comparable postgraduate training contexts should prioritize supervision, organizational clarity, and supportive educational environments alongside scientific productivity.

## Supplementary Information

Supplementary Material 1.

## Data Availability

The datasets used and analyzed during the current study will be made available from the corresponding author on reasonable request.

## Abbreviations

OLBI-SS: Oldenburg Burnout Inventory–Student Version
PHQ-4: Patient Health Questionnaire-4
PSS-4: Perceived Stress Scale-4
GAD-2: Generalized Anxiety Disorder
BRS: Brief Resilience Scale
JD-R: Job Demands–Resources
