# Supplementary material for: The hidden cost of specialization: a mixed-methods study on burnout, mental health, and financial ınstability in dental trainees
Source: BMC Med Educ. 2026 Jan 12;26:229. doi: 10.1186/s12909-026-08584-2 (PMC12888264; doi:10.1186/s12909-026-08584-2)
Supplement: Supplementary file 1 — Supplementary Material 1. [file 12909_2026_8584_MOESM1_ESM.docx]

# Supplementary Table S1. Bootstrapped Regression Estimates (1,000 and 5,000 Resamples)

Bootstrapping was performed to evaluate the stability of regression coefficients. Percentile 95% confidence intervals and standard errors are reported for both 1,000 and 5,000 resampling iterations.

| Predictor | β (1000) | SE (1000) | 95% CI (1000) | p (1000) | β (5000) | SE (5000) | 95% CI (5000) | p (5000) |
| --- | --- | --- | --- | --- | --- | --- | --- | --- |
| PHQ-4 (Mental Health Symptoms) | 1.211 | 0.524 | 0.175 to 2.182 | 0.028 | 1.247 | 0.515 | 0.270 to 2.267 | 0.014 |
| Institutional Support | -3.565 | 1.286 | -5.995 to -1.048 | 0.002 | -3.555 | 1.216 | -5.909 to -1.207 | 0.003 |
| Male Gender | 3.591 | 1.746 | 0.167 to 6.891 | 0.034 | 3.640 | 1.760 | 0.224 to 7.147 | 0.038 |
| Perceived Stress (PSS-4) | 0.06 | 0.05 | -0.71 to 0.82 | 0.412 (ns) | 0.05 | 0.05 | -0.68 to 0.79 | 0.448 (ns) |
| Resilience (BRS) | -2.40 | 2.43 | -5.61 to 0.59 | 0.112 (ns) | -2.43 | 2.39 | -5.54 to 0.52 | 0.103 (ns) |
| Age | -0.16 | 0.20 | -0.69 to 0.36 | 0.542 (ns) | -0.20 | 0.18 | -0.64 to 0.29 | 0.487 (ns) |
| Funding Status (Self-funded) | 1.98 | 1.92 | -4.06 to 7.75 | 0.311 (ns) | 1.92 | 1.88 | -3.98 to 7.58 | 0.322 (ns) |
